# Environmental Risk Factors of Pancreatic Cancer

**DOI:** 10.3390/jcm8091427

**Published:** 2019-09-10

**Authors:** Hui-Jen Tsai, Jeffrey S. Chang

**Affiliations:** 1 National Institute of Cancer Research, National Health Research Institutes, Tainan 70456, Taiwan; 2 Department of Internal Medicine, National Cheng Kung University Hospital, Tainan 70403, Taiwan; 3 Department of Internal Medicine, Kaohsiung Medical University Hospital, Kaohsiung 80756, Taiwan

**Keywords:** environmental risk factors, pancreatic cancer, prevention

## Abstract

Despite the advancement in medical knowledge that has improved the survival rate of many cancers, the survival rate of pancreatic cancer has remained dismal with a five-year survival rate of only 9%. The poor survival of pancreatic cancer emphasizes the urgent need to identify the causes or the risk factors of pancreatic cancer in order to establish effective preventive strategies. This review summarizes the current evidence regarding the environmental (non-genetic, including lifestyle, and clinical factors) risk factors of pancreatic cancer. Based on the current evidence, the established risk factors of pancreatic cancer are cigarette smoking, chronic diabetes, and obesity. Other strong risk factors include low consumption of fruits and vegetables, excess consumption of alcohol, poor oral hygiene, and the lack of allergy history. In the future, more studies are needed to identify additional risk factors of pancreatic cancer, especially the modifiable risk factors that could be included in a public health campaign to educate the public in order to reduce the incidence of pancreatic cancer.

## 1. Introduction

Every year, approximately 460,000 cases (incidence rate = 4.8 per 100,000) of pancreatic cancer are diagnosed worldwide, making it the twelfth most common cancer in the world [1]. Despite advancement in medical knowledge that has improved the survival rate of many cancers, the survival rate of pancreatic cancer has remained dismal with a five-year survival rate of only 9% [2]. As a consequence, the annual number of pancreatic deaths (approximately 430,000 deaths per year with a mortality rate = 4.4 per 100,000) is almost equal to the number of incident cases, making it the cancer with the seventh highest mortality rate in the world [1]. The majority of pancreatic cancers are diagnosed at a late stage. Only 15–20% of pancreatic cancers are resectable with a five-year survival rate of approximately 20% [3,4]. In the era of gemcitabine monotherapy as the standard of systemic primary or adjuvant chemotherapy, the median overall survival (OS) of patients with metastatic, unresectable locally advanced, and resectable pancreatic ductal adenocarcinoma was 5.5–6.5, 13–15, and 22–24 months, respectively, in the randomized phase III trials [5,6,7,8,9,10]. The introduction of a more effective combination of chemotherapy regimens, the FOLFIRINOX (combination chemotherapy that includes folinic acid, fluorouracil (also known as 5FU), irinotecan, and oxaliplatin) and nab–paclitaxel plus gemcitabine combination, improves the median OS of patients with metastatic diseases to 8.5–11.0 months [11,12]. FOLFIRINOX may increase the surgical resection conversion rates and is thus associated with an overall survival of 15.1–18.5 and 21.7–37.7 months in patients with initially borderline resectable and un-resectable locally advanced diseases, respectively [13,14,15,16]. While given as an adjuvant therapy, modified FOLFIRINOX had a 54.4 months overall survival rate in patients with R0/R1 resection in the PRODIGE 24/CCTG PA.6 trial (a multicenter randomized phase III trial comparing FOLFIRINOX vs. gemcitabine as adjuvant therapy for pancreatic cancer) [17]. However, both FOLFIRINOX and nab–paclitaxel plus gemcitabine combination are associated with significant adverse neutropenia events, especially in the Asian population, as compared to gemcitabine monotherapy. The other agents for gemcitabine refractory disease, such as MM398 combined with 5FU/folinic acid or weekly 5FU/folinic acid plus oxaliplatin, are shown to prolong the OS of 1.9 or 2.6 months when compared with the patients who received weekly 5FU/folinic acid. While many other novel agents are under investigation, the overall prognosis of the advanced pancreatic cancer is poor [6,18]. The poor survival of pancreatic cancer emphasizes the urgent need to identify the causes or the risk factors associated with the development of pancreatic cancer in order to establish effective preventive strategies. Furthermore, knowing the risk factors may help identify high-risk individuals for pancreatic cancer screening in order to increase the rate of early diagnosis. The main reason for the abysmal prognosis of pancreatic cancer, in most cases, is due to late stage diagnosis, with approximately 53% showing distant metastasis and 29% having spread to the regional lymph nodes [19]. While the survival of pancreatic cancer overall is very poor, the five-year survival rate can be improved to more than 75% when the tumor is detected and removed at a size less than 10 mm [20].

Many studies have investigated the risk factors of pancreatic cancer. This review summarizes the current evidence regarding the environmental (non-genetic, including lifestyle, and clinical factors) risk factors of pancreatic cancer (Table 1). Many of the environmental risk factors, particularly the lifestyle factors, are modifiable; therefore, knowing the environmental factors influencing the risk of pancreatic cancer may provide opportunities to prevent the occurrence of pancreatic cancer.

## 2. Lifestyle Risk Factors of Pancreatic Cancer

### 2.1. Cigarette Smoking and Other Tobacco Products

Cigarette smoking is a well-recognized risk factor of pancreatic cancer. Approximately 20–25% of pancreatic cancers can be attributed to cigarette smoking [21]. A pooled analysis combined data from 12 case-control studies and reported a positive association between cigarette smoking and pancreatic cancer (former smokers: Odd ratio (OR) = 1.2, 95% confidence interval (CI): 1.0–1.3; current smokers: OR = 2.2, 95% CI: 1.7–2.8) [22]. In addition, the pooled analysis also showed that pancreatic cancer risk could be reduced to the level of never smokers after 20-years of cigarette smoking cessation [22]. Another pooled analysis combining data from eight nested case-control studies reported an increased risk of pancreatic cancer among current cigarette smokers (OR = 1.8, 95% CI: 1.4–2.3) and 15-years of smoking cessation could reduce the risk of pancreatic cancer to the level of never smokers [23]. A dose-response analysis combining data from 78 studies showed that pancreatic risk increased sharply with only a low number of cigarettes per day or just a few years of smoking [24]. The analysis also showed that pancreatic risk decreased rapidly after a few years of smoking cessation, although it may take up to 20-years for the risk to reach the level of never smokers [24]. In addition to cigarette smoking, studies with other tobacco products reported an elevated pancreatic cancer risk associated with cigar smoking, but not with pipe smoking or smokeless tobacco [25].

In contrast to the established association between active smoking and pancreatic cancer, studies on the association between environmental tobacco smoke and pancreatic cancer have been inconclusive. A meta-analysis of data from eight studies reported no significant association between environmental tobacco smoke and pancreatic cancer risk (summary relative risk (RR) = 1.23, 95% CI: 0.86–1.77) [26].

### 2.2. Alcohol Drinking

Studies on the association between alcohol drinking and pancreatic cancer have generated inconsistent results. A positive association was only observed at a high level of alcohol consumption. In a pooled analysis of 14 cohort studies, pancreatic cancer risk increased by approximately 20% for daily consumption of 30 or more grams of alcohol (approximately three cans of beer) (RR = 1.22, 95% CI: 1.03–1.45) [27]. A pooled analysis of 10 case-control studies reported that heavy drinking with nine or more drinks per day was associated with a significantly increased risk of pancreatic cancer (OR = 1.6, 95% CI: 1.2–2.2), while no significant association was found with light to moderate drinking (≤4 drinks/day) [28]. A meta-analysis of 19 cohort studies also found that only high alcohol intake was associated with an increased pancreatic cancer risk [29]. In many East Asian countries, 30–50% of the population carry the ALDH2*2 allele, which is associated with reduced enzyme activity, resulting in the inefficient metabolism of the carcinogenic acetaldehyde generated from ethanol metabolism. Individuals carrying the ALDH2*2 allele have a higher risk of developing alcohol-related cancers [30]. Kanda et al. found that even light to moderate alcohol drinking (<30 g day) may be associated with an increased pancreatic cancer risk among those carrying the ALDH2*2 allele [31]. This suggests that, because of the high percentage of East Asians with inefficient acetaldehyde metabolism, alcohol drinking may play a more significant role in the development of pancreatic cancer among East Asians. Due to the very limited number of studies on the effect modification of the *ALDH2* polymorphism on the association between alcohol drinking and pancreatic cancer, more investigations are needed.

### 2.3. Diet

Numerous studies have investigated the association between diet and pancreatic cancer risk and the results have been inconsistent. Generally, diets that are rich in fruits and vegetables and other plant-based foods have been associated with a reduced pancreatic cancer risk, while dietary patterns rich in meat and animal products have been be associated with an increased pancreatic cancer risk [32,33,34,35,36]. Studies have shown that phytochemicals and dietary fibers contained in plant-based foods, including fruits, vegetables, whole grains, and nuts are beneficial in reducing the risk of cancer [37,38]. Phytochemicals, including carotenoids, phenolics, alkaloids, nitrogen-containing compounds, and organosulfur compounds, have been shown to possess anti-cancer activities [37]. These anti-cancer activities encompass a wide range of mechanisms, including antioxidant properties, anti-inflammatory action, inhibition of the growth, progression, and invasion of cancer cells, and DNA damage repair, etc. Another important component of the plant-based foods is dietary fiber [37]. A meta-analysis combining data from 13 case-control studies and one cohort study reported an inverse association between a higher intake of dietary fiber and pancreatic cancer risk (OR = 0.52, 95% CI: 0.44–0.61), and every 10 grams of daily intake of dietary fiber was associated with a 12% reduction in pancreatic cancer risk (OR = 0.88, 95% CI: 0.84–0.92) [39]. 

### 2.4. Physical Activities

Studies investigating the association between physical activities and pancreatic cancer have generated inconsistent results. A meta-analysis using data from 28 studies found that increasing the level of total and occupational activities were associated with a reduced pancreatic cancer risk [40]. However, the association did not show a dose-response relationship. The largest reduction of pancreatic cancer risk was observed for moderate activity [40]. Another meta-analysis analyzed data from 26 studies and reported that leisure time physical activity was inversely associated with pancreatic cancer risk (RR = 0.89, 95% CI: 0.82–0.96), although the inverse association only occurred in case-control studies (RR = 0.69, 95% CI: 0.59–0.81) and not in cohort studies (RR = 0.96, 95% CI: 0.91–1.02) [41]. Three subsequent studies also produced inconsistent results. A cohort study from USA found no association between physical activity and pancreatic cancer [42]. Another cohort study from Europe reported that physical activity was associated with a reduced pancreatic cancer risk among individuals younger than 60-years (hazard ratio (HR) = 0.27, 95% CI: 0.07–0.99), but not among those older than 60-years (HR = 1.23, 95% CI: 0.96–1.57) [43]. A study analyzed data from two cohort studies in China and reported a reduced pancreatic cancer risk associated with physical activities among men (HR = 0.71, 95% CI: 0.50–1.00) but not among women (HR = 1.06, 95% CI: 0.81–1.38) [44]. Overall, the role of physical activities in preventing the occurrence of pancreatic cancer is inconclusive. However, physical activity, in addition to a healthy diet, is important to prevent obesity, which is a known risk factor of pancreatic cancer. Future studies should consider investigating the interaction between physical activities and diet on the risk of pancreatic cancer. In addition, mediation analysis could be performed to determine the extent to which body weight mediates the effect physical activity has on the risk of pancreatic cancer. 

### 2.5. Obesity

Studies have consistently found a positive association between obesity and an increased risk of pancreatic cancer. A pooled analysis of 2,170 pancreatic cancer cases and 2,209 controls reported an increased pancreatic cancer risk among individuals in the highest quartile BMI group compared to those in the lowest quartile (OR = 1.33, 95% CI: 1.12–1.58) [45]. A meta-analysis with data from 23 cohort studies showed an increased pancreatic cancer risk associated with higher BMI (RR for every five-unit increment = 1.10, 95% CI: 1.07–1.14) [46]. A pooled analysis of 20 prospective cohort studies showed that central obesity was associated with increased pancreatic cancer mortality independent of the baseline BMI [47]. Furthermore, BMI during early adulthood was associated with increased pancreatic cancer mortality later in life [47]. A pooled analysis combining data from nine Japanese cohort studies found a significantly increased pancreatic cancer risk among men, but not among women [48]. A nationwide study of 1.79 million Israeli adolescents reported that adolescent obesity (≥95th percentile) was associated with an increased pancreatic cancer risk later in life compared to normal weight (5th to <85th percentile) for both men (HR = 3.67, 95% CI: 2.52–5.34) and women (HR = 4.07, 95% CI: 1.78–9.29) [49]. The biological mechanisms underlying the association between obesity and pancreatic cancer are not well understood, but inflammation and hormonal misbalance are two possible mediators [50]. More studies are needed to decipher the biological mechanisms that could explain the association between obesity and pancreatic cancer.

### 2.6. Oral Health/Hygiene

Previous studies have consistently reported a significant positive association between poor oral health, including periodontal diseases and tooth loss, and an increased pancreatic cancer risk. In a meta-analysis combining data from eight studies, having periodontitis was significantly associated with an elevated pancreatic cancer risk (RR = 1.74, 95% CI: 1.41–2.15) [51]. A cohort study with 29,104 male smokers reported that tooth loss was associated with an increased pancreatic cancer risk (HR = 1.63, 95% CI: 1.09–2.46) [52]. A study with approximately 40,000 African American women also reported that tooth loss, periodontal disease, or both was associated with an increased pancreatic cancer risk [53]. Overall, studies have concurred on the positive association between poor oral health/hygiene and the risk of pancreatic cancer, although the underlying biological mechanism is yet to be elucidated.

### 2.7. Oral Microbiome 

Poor oral health such as periodontal diseases and tooth loss may be due to an infection from oral pathogenic bacteria. Recent studies have begun to study the role of oral microbiome in the development of pancreatic cancer. Michaud et al. indirectly assessed the association between oral bacteria and pancreatic risk by analyzing the blood antibody levels for oral bacteria in a nested case-control study of 405 pancreatic cancer cases and 416 controls [54]. Their results showed that higher levels of serum antibody for *P. gingivalis* ATTC53978, a periodontopathogenic bacterium, were associated with an elevated pancreatic cancer risk [54]. In addition, they showed that higher levels of antibodies for commensal oral bacteria, which may inhibit the growth of the oral pathogenic bacteria, were associated with a decreased pancreatic cancer risk [54]. Fan et al. collected oral wash samples from 361 pancreatic cancer cases and 371 controls and examined the association between oral bacteria and pancreatic cancer risk by sequencing the bacterial 16S rRNA gene [55]. They reported that individuals with a carriage of oral pathogens, including *P. gingivalis* and *A. actinomycetemcomitans,* had an increased risk of pancreatic cancer [55]. A study by Olson et al. compared the oral bacteria profiles of 40 pancreatic cancer patients, 39 patients with intraductal papillary mucinous neoplasm (IPMN) (a precursor condition with an increased risk of developing into pancreatic cancer), and 58 controls [56]. They found that pancreatic cancer cases had a higher proportion of Firmicutes, whereas controls had a higher proportion of Probacteria; however, these bacteria profiles showed no correlation with measures of oral health [56]. What are the possible explanations for the positive association between oral pathogenic bacteria and pancreatic cancer? 

**Figure 1:**
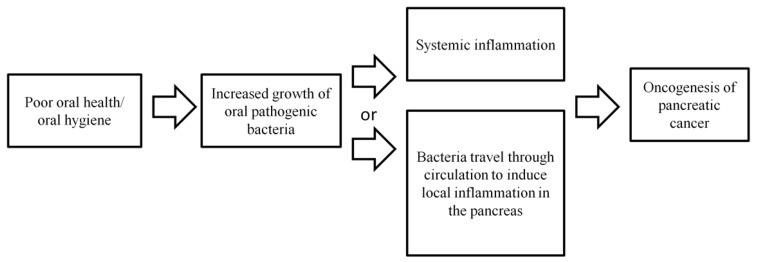
Possible biological mechanisms explaining the association between poor oral health/hygiene and microbiome and an increased pancreatic cancer risk.

It is possible that infection in the oral cavity may promote systemic inflammation, which may include inflammation at a distant site, such as the pancreas (Figure 1). Chronic inflammation, in turn, may promote carcinogenesis [57,58]. Alternatively, oral pathogenic bacteria may be transported to distant sites through circulation and induce local inflammation at the pancreas, leading to an increased risk of developing pancreatic cancer (Figure 1). Overall, the limited numbers of published studies have demonstrated an association between oral bacteria and pancreatic cancer risk. However, the biological role of oral bacteria in the development of pancreatic cancer is inconclusive and more investigations are needed.

### 2.8. Gut Microbiome

Besides oral microbiome, some researchers have also investigated the roles of gut microbiome in the development of pancreatic cancer. Ren et al. compared the gut microbial profiles of 85 pancreatic cancer patients with 57 healthy controls. They observed that some specific pathogens and lipopolysaccharides-producing bacteria were increased in the gut microbial profiles of the pancreatic cancer patients, while probiotics and butyrate-producing bacteria decreased in comparison to the healthy controls [59]. Pushalkar et al. showed the capacity of gut bacteria to migrate to the pancreas, suggesting the possibility of a direct interaction between gut bacteria and the microenvironment of the pancreas [60]. In addition, they showed that pancreatic cancer harbors more bacteria than a normal pancreas [60]. Mendez et al. observed microbial dysbiosis in the early stage of pancreatic cancer oncogenesis in the pancreatic cancer mouse model, suggesting the possibility of measuring gut microbes as biomarkers for the early detection of pancreatic cancer [61]. The current information regarding the association between gut microbiome and pancreatic cancer is very limited and more investigations are required. In addition, several of the pancreatic cancer-associated factors, including cigarette smoking, obesity, heavy alcohol drinking, diabetes, chronic pancreatitis, and allergies, have been associated with alterations in gut microbiome [62]. It would be important to determine whether gut microbiome is a mediator for these other environmental factors in the oncogenesis of pancreatic cancer or has an independent influence on the development of pancreatic cancer. 

### 2.9. Coffee 

Many studies have investigated the association between coffee and pancreatic cancer and the results have been inconsistent. Most of these studies were case-control studies that may suffer from biases of recall and control selection. Recent larger cohort studies have reported a null association between coffee consumption and pancreatic cancer. Zhou et al. analyzed the data of 309,797 never-smoking women with a median follow-up time of 13.7-years from the UK prospective Million Women Study and found no significant association between coffee drinking and pancreatic cancer risk (RR for 1–2 daily cups: 1.02, 95% CI: 0.83–1.26; RR for 3–4 daily cups: 0.96, 95% CI: 0.76–1.22; and RR for 5 or more daily cups: 0.87, 95% CI: 0.64–1.18) [63]. They further combined their data with data from three other cohort studies in a meta-analysis and reported a null association between daily drinking of two or more cups of coffee and pancreatic cancer risk (RR = 1.00, 95% CI: 0.86–1.17) [63]. Guertin analyzed data of 457,366 US adults from the US NIH-AARP Diet and Health Study with a total follow-up time of more than 4,155,256 person-years and observed no significant association between coffee consumption and pancreatic cancer risk (RR for <1 daily cup: 1.05, 95% CI: 0.85–1.30; RR for 1 daily cup: 1.06, 95% CI: 0.86–1.31; RR for 2–3 daily cups: 1.03, 95% CI: 0.85-1.25; RR for 4–5 daily cups: 1.00, 95% CI: 0.79–1.25; and RR for 6 or more daily cups: 1.24, 95% CI: 0.93–1.65) [64].

## 3. Clinical Risk Factors of Pancreatic Cancer

### 3.1. Diabetes

**Figure 2:**
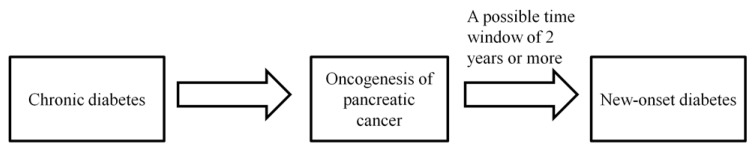
Diabetes can be both a cause and a consequence of pancreatic cancer.

Diabetes is a well-established risk factor of pancreatic cancer (Figure 2). A pooled analysis combining data from 15 case-control studies showed that having diabetes for >2-years was associated with an elevated pancreatic cancer risk (OR = 1.90, 95% CI: 1.72–2.09), and the increased risk even persisted for diabetes with a duration of >20-years (OR = 1.30, 95% CI: 1.03–1.63) [65]. A meta-analysis of 23 cohort studies showed that previously diagnosed diabetes was associated with a 52% increase in pancreatic cancer risk (HR = 1.52, 95% CI: 1.43–1.63) [66]. Overall, studies have concurred that long-term diabetes is a risk factor of pancreatic cancer. 

Besides being a risk factor, diabetes may also be a consequence of pancreatic cancer. Chari et al. showed that new-onset diabetes might occur as early as two-years before the diagnosis of pancreatic cancer [67] (Figure 2). A cohort study of 48,995 African Americans and Latinos showed that although both recent onset (<3-years) diabetes and long-term diabetes were associated with an increased pancreatic cancer risk, recent onset diabetes was associated with a 2.3 times greater increase in pancreatic cancer risk than long-term diabetes, thus supporting that recent onset diabetes may be an indicator for the occurrence of pancreatic cancer [68].

### 3.2. Chronic Pancreatitis

Studies have indicated a strong positive association between chronic pancreatitis and pancreatic cancer. A meta-analysis combining data from 13 studies showed that chronic pancreatitis was strongly associated with an increased risk of pancreatic cancer diagnosed within two-years of chronic pancreatitis (RR = 16.16, 95% CI: 12.59–20.73); however, the association diminished in strength when the lag-time between chronic pancreatitis and pancreatic cancer was set at five-years (RR = 7.90, 95% CI: 4.26–14.66) and nine-years (RR = 3.53, 95% CI: 1.69–7.38) [69], suggesting that part of the association could be due to reverse causality and the role of chronic pancreatitis in the occurrence of pancreatic cancer might be weaker than expected. It has been estimated that patients with chronic pancreatitis have only a 5% lifetime risk of developing pancreatic cancer [70]. This suggests that, although chronic pancreatitis is associated with an elevated pancreatic cancer risk, it likely only accounts for a small percentage of pancreatic cancer cases.

### 3.3. Allergies

**Figure 3:**
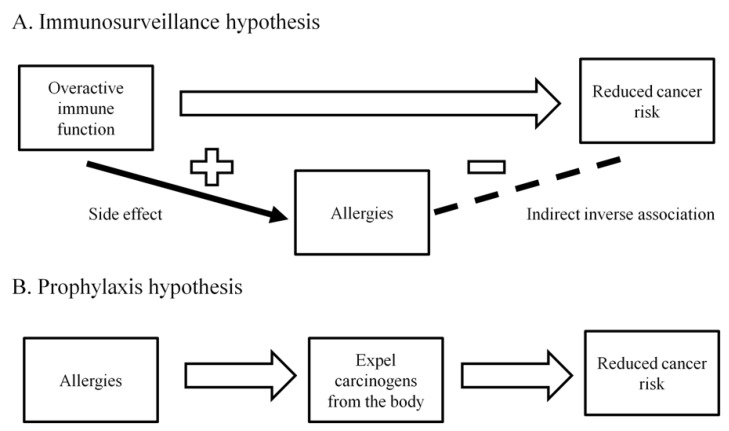
A. Immunosurveillance hypothesis: Allergy does not play a direct role in preventing the occurrence of cancer, but is merely a manifestation of an overactive immune function that has a high efficiency of surveying and eradicating cancer cells. B. Prophylaxis hypothesis: Allergic reactions are the body’s method of expelling the carcinogens.

Studies have often reported an inverse association between allergy and pancreatic cancer risk. In 2005, a meta-analysis combining data from 14 studies showed that having any allergic symptoms was associated with a decreased pancreatic cancer risk (RR = 0.82, 95% CI: 0.68–0.99) [71]. A pooled analysis of 10 case-control studies reported that having any allergy (OR = 0.79, 95% CI: 0.62–1.00), hay fever (OR = 0.74, 95% CI: 0.56–0.96), or allergy to animals (OR = 0.62, 95% CI: 0.41–0.94) were all associated with a lower pancreatic cancer risk [72]. A cohort study of 187,226 subjects with an average follow-up time of 16-years observed a null association between allergy and pancreatic cancer incidence (RR = 1.00, 95% CI: 0.88–1.12), although among individuals aged 70-year or older, an inverse association between allergy and pancreatic cancer incidence was reported (RR = 0.74, 95% CI: 0.56–0.98) [73]. More cohort studies are needed to confirm the association observed by the majority of case-control studies between allergy and a reduced pancreatic cancer risk. In addition, the biological mechanism underlying this inverse association needs to be deciphered in order to determine whether this can be translated into a strategy to prevent the occurrence of pancreatic cancer. Two hypotheses, the “immunosurveillance hypothesis” and the “prophylaxis hypothesis”, have been proposed to explain the inverse association between allergy and various cancers [74]. The “immunosurveillance hypothesis” states that allergy does not play a direct role in preventing the occurrence of cancer, but is merely a manifestation of an overactive immune function that has a high efficiency of surveying and eradicating cancer cells [74] (Figure 3A). In contrast, the “prophylaxis hypothesis” states that allergy is directly involved in cancer prevention because allergic reactions are the body’s method of expelling the carcinogens [74,75] (Figure 3B). How the inverse association between allergy and pancreatic cancer fits into these two hypotheses requires further investigations.

### 3.4. Infections

Several infections, including hepatitis B and C and *Helicobacter pylori* (*H. pylori*), have been examined in regards to their association with pancreatic cancer. A meta-analysis of eight studies reported that both hepatitis B and C infections were associated with an elevated risk of pancreatic cancer [76]. However, one subsequent cohort study from Japan reported no association between either hepatitis B or C and pancreatic cancer [77]. A retrospective cohort study from Taiwan reported a significantly increased pancreatic cancer risk associated with hepatitis B, while the association with hepatitis C was not statistically significant [78]. A case-control study from Taiwan measured the blood markers for hepatitis B and C infections among 585 pancreatic cancer cases and 1,716 controls and found no association between hepatitis B or C and the risk of pancreatic cancer after adjusting for age, sex, diabetes, and smoking [79]. In a cohort study of 12,126 patients with chronic hepatitis, hepatitis C infection was associated with a significantly higher incidence of pancreatic cancer [80]. Overall, the majority of studies suggested that hepatitis B or C infection may be associated with an increased pancreatic cancer risk.

In a meta-analysis combining data from 10 studies, *H. pylori* seropositivity showed no overall association with pancreatic risk; however, seropositivity for CagA-positive *H. pylori* was associated with a reduced pancreatic cancer risk (OR = 0.78, 95% CI: 0.67–0.91), while seropositivity for CagA-negative *H. pylori* was associated with an increased pancreatic cancer risk (OR = 1.30, 95% CI: 1.02–1.65) [81]. The differential risk of pancreatic cancer according to the strain of *H. Pylori* has yet to be confirmed by additional studies. A nested case-control study published after the meta-analysis reported seropositivities for *H. pylori* overall, for CagA-positive *H. pylori,* and for CagA-negative *H. Pylori* were all not associated with pancreatic cancer risk [82]. More studies on the association between the different strains of *H. pylori* and pancreatic cancer are needed to confirm the strain-specific association between *H. pylori* and pancreatic cancer.

## 4. Incorporation of Environmental Risk Factors for Constructing Pancreatic Cancer Risk Prediction Models

One of the important purposes of identifying the risk factors of pancreatic cancer is to construct pancreatic cancer risk prediction models to identify high-risk individuals for screening in order to increase early diagnosis and improve survival rates. Several study groups have constructed pancreatic cancer risk prediction models with environmental risk factors alone or in combination with laboratory biomarkers or genetic polymorphisms. Klein et al. constructed a pancreatic cancer prediction model based on data from 3,349 pancreatic cancer patients and 3,654 controls [83]. The variables included in their risk model were current smoking, heavy alcohol use (more than 3 drinks per day), BMI > 30 kg/m^2^, history of diabetes >3-years, family history of pancreatic cancer, non-O ABO genotype, and three single nucleotide polymorphisms (SNPs) (1q32 rs3790844, 5p15 rs401681, 13q22 rs9543325) [83]. The area under the curve (AUC) for their prediction model was 0.61 [83]. Risch et al. constructed a pancreatic cancer prediction model by combining data from 362 pancreatic cancer cases and 690 controls with the 2008–2010 incidence data from US surveillance epidemiology and end results [84]. Their model included current cigarette smoking, current use of proton pump-inhibitor anti-heartburn medications, recent diagnosis of diabetes, recent diagnosis of pancreatitis, Jewish ancestry, and the non-O ABO blood group [84]. They were able to calculate the five-year absolute risk of pancreatic cancer based on the different combinations of risk factors with some of the five-year risks reaching 5–10%, which may warrant pancreatic cancer screening [84]. Yu et al. constructed sex-specific pancreatic cancer prediction models using data from a cohort of 1,289,933 Korean men and 557,701 Korean women with a validation cohort of 500,046 Korean men and 627,629 Korean women [85]. The prediction model for men included age, height, BMI, fasting glucose, urine glucose, smoking, and age of smoking initiation [85]. The prediction model for women included height, BMI, fasting glucose, urine glucose, smoking, and alcohol drinking. The models showed good discrimination ability with an AUC of 0.813 for men and 0.804 for women [85]. Using data from 664 Japanese pancreatic cancer cases and 664 age and sex-matched Japanese controls, Nakatochi et al. constructed two pancreatic cancer risk prediction models with cigarette smoking, family history of pancreatic cancer, and five SNPs [86]. The full model with all seven factors had an AUC of 0.63, while the model with only the five SNPs had an AUC of 0.61, suggesting an advantage of including both environmental and genetic factors in the risk prediction model [86]. As new-onset diabetes may be a consequence of the development of pancreatic cancer, individuals with new-onset diabetes have been considered as candidates for pancreatic cancer screening. Chari et al. compared the prevalence of diabetes between 736 pancreatic cancer cases and 1,875 controls and found that the prevalence of diabetes became significantly different at three-years prior to the cases’ pancreatic cancer diagnosis [67]. This suggests that pancreatic cancer-associated diabetes may occur as early as three-years prior to the diagnosis of pancreatic cancer and screening patients with new-onset diabetes may therefore provide a sufficient window of opportunity for the early diagnosis of pancreatic cancer at an operable stage. However, because the majority of new-onset diabetes cases are type-2 diabetes, screening every new-onset diabetes case for pancreatic cancer is not cost-effective. In addition to new-onset diabetes, other risk factors or clinical parameters are needed to improve the accuracy of the pancreatic cancer prediction model. Boursi et al. constructed a pancreatic cancer prediction model using data from 109,385 new-onset diabetes patients [87]. The model, which has an AUC of 0.82, included age, BMI, change in BMI, smoking, use of proton pump inhibitors, anti-diabetic medications, and several biomarkers, including haemoglobin A1C, cholesterol, haemoglobin, creatinine, and alkaline phosphatise [87]. If the risk of developing pancreatic cancer were set at 1% over three-years of follow-up, only 6.2% of the new-onset diabetes would need to be screened for pancreatic cancer, achieving a sensitivity of 44.7%, a specificity of 94%, and a positive predictive value of 2.6% [87]. While several pancreatic cancer prediction models have been proposed, validation studies and cost-effective analysis for these models need to be performed. In the future, more investigations are needed to search for risk factors and biomarkers that may improve the accuracy of the pancreatic cancer prediction model.

## 5. Conclusions

Based on the current evidence, the current recommendations to reduce the risk of pancreatic cancer include cessation of cigarette smoking, diabetes control, and maintenance of an ideal body weight (Table 2 summarizes the key points for each of the environmental factors discussed in this paper). Other actions that may be beneficial for pancreatic cancer prevention include a diet rich in vegetables and fruits, good oral hygiene practice, and avoidance of excess alcohol consumption. While many studies have investigated the association between various environmental risk factors and pancreatic cancer, only a few environmental risk factors have shown strong and consistent evidence. In the future, more studies are needed to identify additional risk factors of pancreatic cancer, especially the modifiable risk factors that can be included in a public health campaign to educate the public in order to reduce the incidence of pancreatic cancer. Furthermore, the identification of additional risk factors may improve the accuracy of the pancreatic cancer risk prediction model to identify high-risk individuals for pancreatic cancer screening in order to increase the probability of early diagnosis and to improve patient outcomes.

## Figures and Tables

**Table 1:** Environmental (non-genetic, including lifestyle, and clinical factors) risk factors of pancreatic cancer by the level of evidence.

The Level of Evidence	Risk Factors
Very strong	Cigarette smoking, chronic diabetes, obesity
Strong	Poor oral health/oral hygiene, chronic pancreatitis, no history of allergies, heavy alcohol drinking, dietary patterns with low amount of vegetables and fruits
Moderate, although further investigations are required	Dietary patterns rich in meat and animal products, oral/gut microbiome, hepatitis B or C infection
The level of evidence is unclear, further investigations are required	Environmental tobacco smoke, light to moderate alcohol drinking, physical inactivity, coffee, *Helicobacer pylori* infection

**Table 2:** Summary of key points for each of the environmental factors discussed in this paper.

Environmental Factors	Key Points
Cigarette smoking	Cigarette smoking is an established risk factor of pancreatic cancer. Smoking cessation can reverse the increased risk of pancreatic cancer associated with cigarette smoking
Alcohol drinking	Heavy alcohol use is associated with an increased pancreatic cancer risk. The association between low to moderate level of alcohol use and pancreatic cancer is unclear.
Diet	Diet rich in fruits and vegetables and other plant-based foods has been associated with a reduced pancreatic cancer risk
Physical activities	The role of physical activities in preventing the occurrence of pancreatic cancer is inconclusive, although they may possibly reduce the pancreatic cancer risk by preventing obesity.
Obesity	Obesity is an established risk factor of pancreatic cancer, although biological mechanisms are yet to be delineated
Oral health/hygiene and oral microbiome	Poor oral health/hygiene has consistently been associated with an increased pancreatic cancer risk. Poor oral health/hygiene may affect oral microbiome by promoting the growth of pathogenic oral bacteria, which may increased the risk of pancreatic cancer by inducing inflammation and other mechanisms. More investigations are needed for the role of oral microbiome in the development of pancreatic cancer
Gut microbiome	The current information regarding the association between gut microbiome and pancreatic cancer is very limited and more investigations are required
Coffee	The association between coffee and pancreatic cancer is unclear
Diabetes	Diabetes can be both a risk factor and a consequence of pancreatic cancer
Chronic pancreatitis	Chronic pancreatitis is a known risk factor of pancreatic cancer with a lifetime risk of 5%
Allergies	Allergies are associated with a reduced pancreatic cancer risk, although the biological mechanisms are unclear
Infections	The majority of studies suggested that hepatitis B or C infection may be associated with an increased pancreatic cancer risk. More studies on the association between the different strains of *H. Pylori* and pancreatic cancer are needed to confirm the strain-specific association between *H. Pylori* and pancreatic cancer.

## Abbreviations

AUC	Area under the curve
CI	confidence interval;
IPMN	intraductal papillary mucinous neoplasm;
HR	hazard ratio;
OS	overall survival;
OR	odds ratio;
RR	relative risk;
SNP	Single nucleotide polymorphism
